# Data to clarify the landfill role in the case of groundwater quality degradation (Southern Italy)

**DOI:** 10.1016/j.dib.2018.08.201

**Published:** 2018-09-07

**Authors:** Livia Emanuela Zuffianò, Pier Paolo Limoni, Giorgio De Giorgio, Maurizio Polemio

**Affiliations:** Istituto di Ricerca per la Protezione Idrogeologica – CNR, Via Amendola, 122 I, Bari, Italy

## Abstract

The data presented in this article are related to the research article described by “How can the role of leachate on nitrate concentration and groundwater quality be clarified? An approach for landfills in operation (Southern Italy)” (Cossu et al., 2018).

The data set for this article contains chemical analyses of groundwater and leachate, isotope analysis of groundwater and leachate around a group of landfills located in the municipality of Conversano, close to Bari, the main town of the Apulia Region (Southern Italy).

Groundwater samples were collected from eighteen wells.

The hydrogeological and chemical study was used to define geochemical features, groundwater and leachate characteristics and to study their potential macroscopic mixing.

The land use analysis highlighted quantity and type of used fertilizers permitting to compare these with groundwater in terms of isotopic signature.

**Specifications table**

**Table:** 

Subject area	*Earth and Planetary Science*
More specific subject area	*Environmental Science, hydrogeology, geochemistry, isotopic*
Type of data	*Table, figure*
How data was acquired	*pH, EC, T, TDS, Eh, and DO (multi-parametric probe Quanta G Hydrolab model);*
	*Li*^*+*^*, Ca*^*2+*^*, Mg*^*2+*^*, K, Na, Fe and Mn (ICP-OES spectrometry);*
	*F*^*−*^*, Cl*^*−*^*, SO*_*4*_^*2*^^*−*^*, NO*_*3*_^*−*^*and NH4*^*+*^*(ion chromatography);*
	^*3*^*H (liquid scintillation counting, Perkin Elmer Quantulus GCT 6220 Liquid Scintillation Analyzer);*
	*Water-level tape, subcentimeter graduated;*
	*Standard rain and temperature gauge;*
Data format	*Analyzed.*
Experimental factors	*Sampling procedures included: 1) measuring in the field of EC, T, pH, TDS, Eh, and DO; 2) assessing the alkalinity in the field, by means of titration with HCl; 3) acidifying samples for cation analysis by the addition of HNO*_*3*_*to a pH < 2.*
	*Water samples for metals determination was not filtered but was acidified before chemical analysis. This choice was due to the scope to explain some anomalously high iron contents that were detected in previous determinations in the area.*
	*Water sampling and storing*^*3*^*H determinations do not require any specific pre-treatment.*
Experimental features	*Determination of physical, chemical and isotopic parameters with the purpose to clarify the role of landfill leakage on groundwater quality degradation.*
Data source location	*Conversano, Italy*
Data accessibility	*Data are available in the article*

**Value of the data**

• The data could be used to determine groundwater quality and the level of chemical contamination due to a group of landfills.
• The data clarify the lack of landfill role in the case of nitrate groundwater quality degradation, highlighting the role of fertilizers.
• The data could be helpful for concerned authorities and policy makers in water quality management.

## 1 Data

**Fig. 1:**
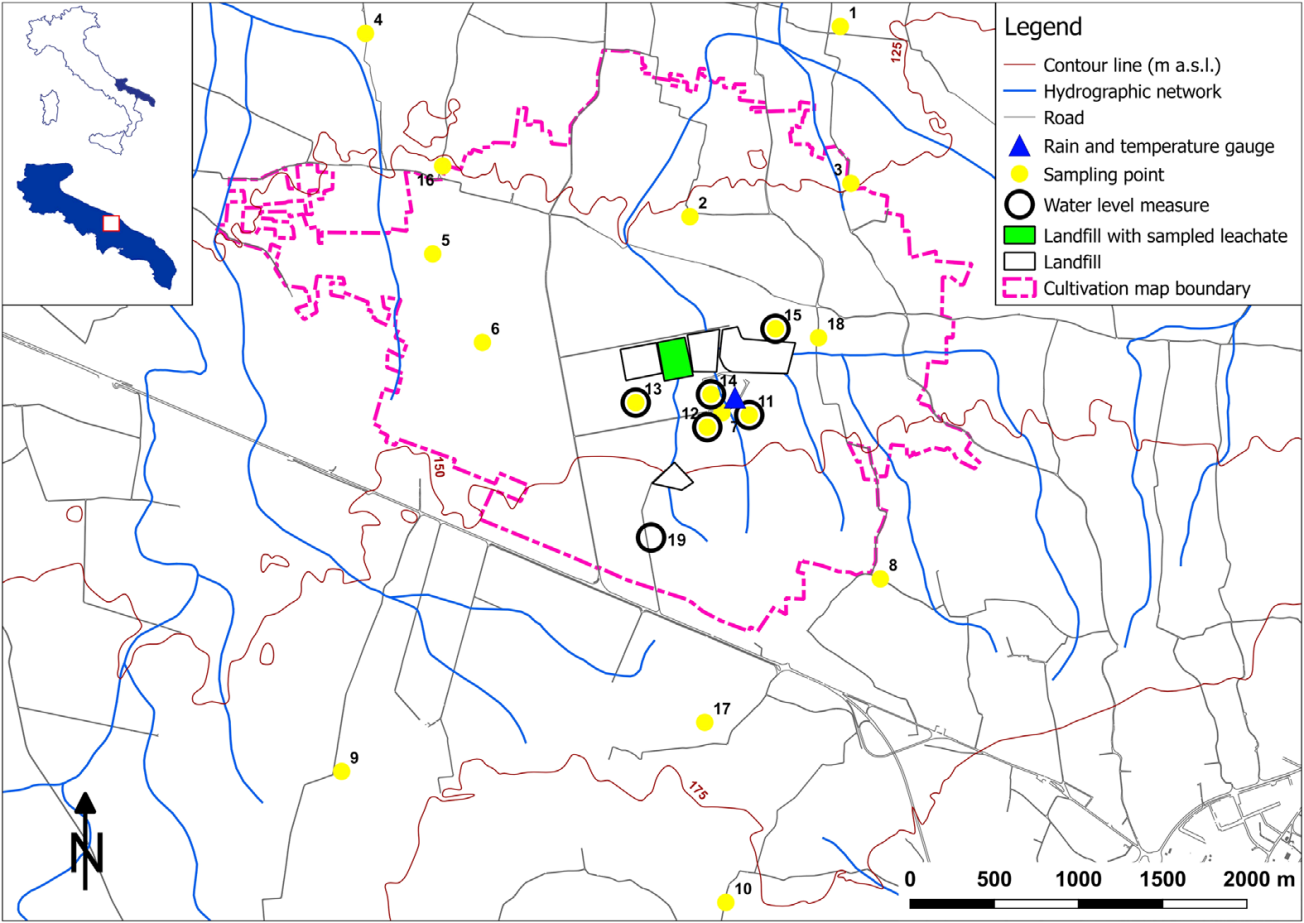
General survey map: survey I, sampling of November 2014 (Table 2); cultivation map boundary (Table 5); rain and temperature gauge (Table 6); and piezometric measurements (Table 7).

**Fig. 2:**
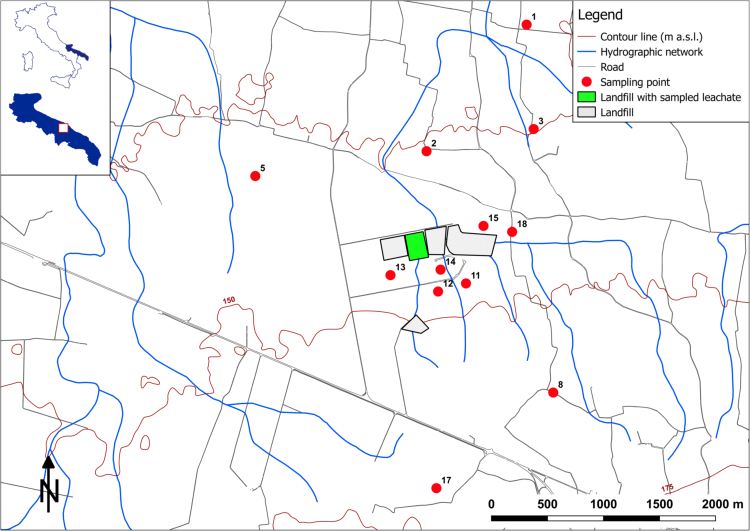
Survey map: survey II, sampling of March 2015 (Table 3); cultivation map boundary (Table 5).

**Fig. 3:**
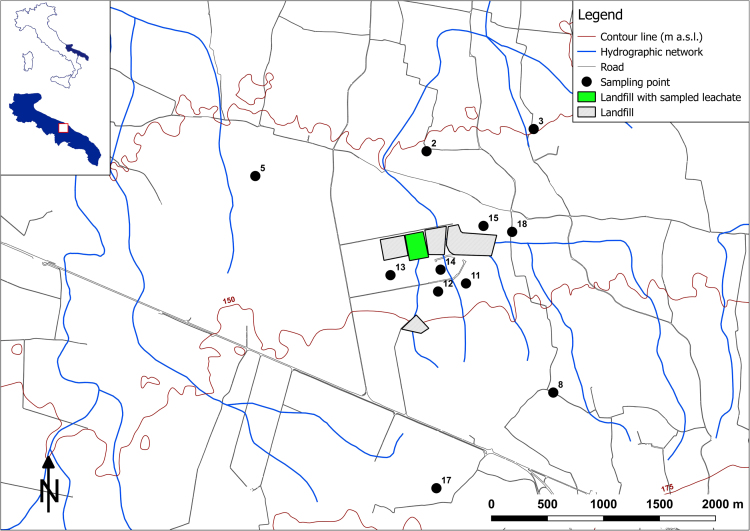
Survey map: survey III, sampling of June 2015 (Table 4); cultivation map boundary (Table 5).

The whole research experience is described by Cossu et al. [1]; in these context, the authors of this paper managed the whole set of on-site measurements and/or sampling. The data contains geochemical and isotopic analysis of groundwater samples collected in eighteen (18) wells (Table 1) located close to a group of landfills and the leachate of one landfill (Fig. 1). Surveys were realized during November 2014 (Fig. 1 and Table 2), March (Fig. 2 and Table 3) and June 2015 (Fig. 3 and Table 4).

**Table 1:** Well sampling point. Datum WGS 84 (UTM). Un. = unknown.

**ID**	**Nord**	**Est**	**Altitude (m a.s.l.)**	**Well depth (m)**
1	4543033	675291	119.0	300
2	4541904	674398	130.0	un.
3	4542102	675354	125.0	277
4	4542992	672471	120.0	281
5	4541683	672870	133.0	289
6	4541157	673165	140.0	347
7	4540739	674588	144.0	328
8	4539752	675529	162.0	452
9	4538607	672329	164.0	462
10	4537829	674611	177.0	368
11	4540725	674750	146.9	198
12	4540653	674501	144.3	250
13	4540799	674076	142.1	349
14	4540848	674523	141.5	250
15	4541244	674904	141.0	250
17	4538899	674486	170.0	320
18	4541185	675162	139.0	365
19	4539995	674175	158.5	250

**Table 2:** Values of the physical and chemical parameters sampled in November 2014 (Survey I). Sampling points in Fig. 1; n.d. = not determined.

		**1**	**2**	**3**	**4**	**5**	**6**	**7**	**8**	**9**	**10**	**Leachate**
**E.C.**	mS/m	2.91	1.59	1.054	1.128	0.871	2.15	1.124	0.819	0.721	0.793	26.5
**T**	°C	17.04	16.80	16.04	17.09	16.74	16.90	17.10	16.77	17.03	17.21	n.d.
**pH**	-	7.04	7.10	7.15	7.12	7.09	7.27	7.12	7.29	7.27	7.11	8.20
**D.O.**	mg/L	3.14	4.19	6.03	5.52	4.86	5.68	6.98	5.02	6.58	4.51	n.d.
**Eh**	mV	116	215	123	157	142	129	89	110	242	147	n.d.
**TOC**	mg/L	1.2	2.2	0.6	0.5	1.1	0.9	0.5	0.5	0.6	0.5	1247.0
**BOD**_**5**_	mg O_2_/L	2.1	1.0	1.0	1.0	3.2	9.3	5.4	4.9	7.3	4.9	410.0
**COD**	mg/L	51.2	5.0	12.5	7.5	10.0	5.0	<2.5	<2.5	<2.5	10.0	5210.0
**Li**^**+**^	µg/L	15.9	8.8	6.3	5.3	4.2	8.4	5.3	5.3	4.2	4.2	22.4
**Na**^**+**^	mg/L	333.8	140.0	39.9	78.8	32.6	239.4	62.0	27.3	18.9	36.8	1720
**N-NH**_**4**_^**+**^	mg/L	<0.1	<0.1	<0.1	<0.1	<0.1	<0.1	<0.1	<0.1	<0.1	<0.1	2051.0
**K**^**+**^	mg/L	8.8	3.8	1.1	3.2	2.1	7.4	1.1	3.2	10.5	20	1239.4
**Ca**^**2+**^	mg/L	141.3	113.8	133.4	101.9	101.9	111.3	122.9	91.4	100.8	94.5	55.5
**Mg**^**2+**^	mg/L	105.0	70.0	31.5	54.6	53.6	84.0	57.8	57.8	49.4	54.6	32.6
**Cl**^**-**^	mg/L	848.0	368.7	105.3	205.7	78.1	535.3	152.0	53.5	22.6	34.3	3788
**F**^**-**^	mg/L	0.15	1.0	0.15	0.11	0.14	0.1	0.1	0.13	0.14	0.24	3.1
**SO**_**4**_^**2-**^	mg/L	94.7	40.4	13.8	25.8	11.7	52.7	26.4	13.0	6.7	10.3	18.7
**HCO**_**3**_^**-**^	mg/L	378.3	378.3	390.5	402.7	402.7	451.5	475.9	475.9	439.3	475.9	12813.8
**NO**_**3**_**-**	mg/L	14.6	31.3	59.9	30.2	32.5	19.1	37.5	13.4	22.5	23.7	115.0
**Fe**^**2+**^	µg/L	79.6	16.6	55.5	11.1	11.4	13.1	30.2	45.0	21.4	30.8	1232.6
**Mn**^**2+**^	µg/L	0.8	0.2	0.5	0.3	0.3	0.3	4.1	0.1	0.1	0.1	41.6
**Tritium**	TU	<0.60	<0.60	0.90±0.63	<0.60	1.10±0.79	0.90±0.62	1.40±0.59	<0.60	0.60±0.66	0.70±0.68	235.00±9
**d-excess**	(‰)	13.87	13.99	12.33	13.76	13.60	14.19	12.71	12.96	11.26	11.75	48.34

		**11**	**12**	**13**	**14**	**15**	**16**	**17**	**18**	**Leachate**
**E.C.**	mS/m	0.875	0.954	1.426	1.66	0.973	0.784	0.762	0.957	26.5
**T**	°C	16.61	16.62	17.00	17.24	17.02	16.78	16.45	17.17	n.d.
**pH**	-	7.21	7.11	7.07	7.02	7.13	7.22	7.11	7.34	8.20
**D.O.**	mg/L	8.25	5.42	5.29	6.78	6.58	5.04	5.05	5.94	n.d.
**Eh**	mV	132	24	101	106	56	135	161	-52	n.d.
**TOC**	mg/L	0.7	0.8	0.8	1.0	2.9	0.8	1.2	1.9	1247.0
**BOD**_**5**_	mg O_2_/L	7.6	9.3	6.0	9.8	3.2	3.8	3.8	2.7	410.0
**COD**	mg/L	5.0	10.0	5.0	<2.5	<2.5	<2.5	10.0	<2.5	5210.0
**Li**^**+**^	µg/L	4.2	4.4	6.3	7.4	3.2	7.4	4.2	5.3	22.4
**Na**^**+**^	mg/L	18.9	38.5	104.0	135.5	20.0	26.4	15.8	36.8	1720
**N-NH**_**4**_^**+**^	mg/L	<0.1	<0.1	<0.1	<0.1	<0.1	<0.1	<0.1	<0.1	2051.0
**K**^**+**^	mg/L	2.1	7.7	5.3	13.7	1.1	11	5.3	2.1	1239.4
**Ca**^**2+**^	mg/L	87.2	119.9	123.9	149.1	149.1	149.1	149.1	149.1	55.5
**Mg**^**2+**^	mg/L	43.1	55.0	63.0	65.1	53.6	39.6	48.3	46.2	32.6
**Cl**^**-**^	mg/L	56.5	81.0	277.9	370.5	146.6	48.7	29.1	104.5	3788
**F**^**-**^	mg/L	0.11	0.13	0.12	0.1	0.13	0.16	0.14	0.4	3.1
**SO**_**4**_^**2-**^	mg/L	16.7	19.5	34.3	44.9	5.5	17.3	12.2	13.9	18.7
**HCO**_**3**_^**-**^	mg/L	463.7	524.7	512.5	524.7	402.7	414.9	475.9	439.3	12813.8
**NO**_**3**_**-**	mg/L	41.2	36.7	36.5	40.9	42.2	40.7	21.8	26.2	115.0
**Fe**^**2+**^	µg/L	10.3	861.0	10.4	12.0	305.3	1543.5	49.5	249.0	1232.6
**Mn**^**2+**^	µg/L	0.3	0.3	0.2	0.3	2.5	0.3	0.3	0.8	41.6
**Tritium**	TU	1.30±0.82	1.40±0.74	0.90±0.70	2.00±0.96	2.30±0.65	1.20±0.68	0.90±0.70	1.40±0.59	235.00±9
**d-excess**	(‰)	11.16	12.27	11.17	13.21	11.56	12.53	13.44	12.74	48.34

**Table 3:** Values of the physical and chemical parameters sampled in March 2015 (Survey II). Sampling points in Fig. 2; n.d. = not determined.

		**1**	**2**	**3**	**5**	**8**	**11**	**12**	**13**	**14**	**15**	**17**	**18**	**Leachate**
**E.C.**	mS/m	1.51	0.989	0.807	0.719	0.684	0.74	0.979	1.452	1.66	0.945	0.755	0.947	21.8
**T**	°C	16.91	16.7	12.74	16.36	16.65	16.56	16.55	16.98	17.15	17	16.37	12.07	n.d.
**pH**	–	7.01	6.93	6.97	6.97	6.92	7.06	6.77	6.82	6.73	6.92	6.82	7.18	7.9
**D.O.**	mg/L	3.16	3.63	4.39	4.22	3.87	3.58	3.19	2.84	2.46	3.13	3.54	2.72	n.d.
**Eh**	mV	113	196	119	214	192	141	55	179	126	104	182	161	n.d.
**TOC**	mg/L	3.3	2.5	2.9	3.3	2.8	2.4	2.2	2.1	2.1	3.2	2	2.8	970
**BOD**_**5**_	mg O_2_/L	7.6	3.2	10	6	2.7	1	1	1	1.6	1	1	1	335
**COD**	mg/L	10	5	10	7.5	5	5	7.5	10	10	5	10	5	2240
**Li**^**+**^	µg/L	22.1	10.5	11.6	7.8	6.7	8.9	8.9	13.8	15.1	6.1	8.4	8.4	29.0
**Na**^**+**^	mg/L	167.5	68.9	5.9	22.1	21.7	24.8	38.2	118.8	136.3	20.5	15.4	34	2162.9
**NH**_**4**_^**+**^	mg/L	<0.1	<0.1	<0.1	<0.1	<0.1	<0.1	<0.1	<0.1	<0.1	<0.1	<0.1	<0.1	1603
**K**^**+**^	mg/L	7.5	3.7	2	1.1	5.5	3.8	2.2	8.7	6.8	2.3	1.8	1.9	1468.8
**Ca**^**2+**^	mg/L	117.8	108.2	131.7	99.6	96.9	111.9	108.7	115.7	138.2	114.6	98.5	105	49.5
**Mg**^**2+**^	mg/L	70.3	53	25.9	45.5	42.6	48.8	53.6	63.9	65.8	52	47.1	45.7	51.8
**Cl**^**−**^	mg/L	308	103.5	84.1	43.7	35.4	45.1	66.3	215	239.3	105.5	25.9	72.8	2725.6
**F**^**−**^	mg/L	<0.1	0.1	<0.1	0.2	0.2	<0.1	0.1	<0.1	0.2	<0.1	0.1	<0.1	0.9
**SO4**_**2**_^**−**^	mg/L	36.6	20.9	11.3	10.4	10.5	15.9	17.4	31	34.3	3.9	10.8	11.9	58.8
**HCO**_**3**_^**−**^	mg/L	463.7	439.3	390.5	439.3	439.3	475.9	549.2	518.7	518.7	457.6	475.9	475.9	10,006
**NO**_**3**_**-**	mg/L	15.8	44.2	57.1	34.9	26.4	38.8	35	31.5	37.3	36.2	20.8	22.5	5.9
**Fe**^**2+**^	µg/L	37.8	8.7	71.8	67.4	25.1	9.9	871.7	6.8	88.2	170.6	58.8	1785.0	5454.0
**Mn**^**2+**^	µg/L	3.4	0.2	3.2	0.5	0.8	0.5	10.5	0.3	4.6	5.3	0.9	21.5	47.7
**Tritium**	TU	<0.6	1.0 ± 0.75	0.7 ± 0.73	2.1 ± 0.47	1.0 ± 0.50	2.0 ± 0.38	2.0 ± 0.64	1.6 ± 0.54	2.2 ± 0.48	3.1 ± 0.48	1.1 ± 0.51	2.2 ± 0.66	182 ± 8.6
**d-excess**	(‰)	13.69	12.67	11.7	12.25	12.29	12.25	14.66	13.21	13.89	13.22	14.19	13.4	39

**Table 4:** Values of the physical and chemical parameters sampled in June 2015 (Survey III). Sampling points in Fig. 3; n.d. = not determined.

		**2**	**3**	**5**	**8**	**11**	**12**	**13**	**14**	**15**	**17**	**18**	**leachate**
**E.C.**	mS/m	1.530	1.031	1.033	0.792	0.874	0.952	1.550	1.820	0.964	0.760	1.580	24.8
**T**	°C	16.99	19.48	17.34	16.92	16.67	17.15	17.09	17.30	17.73	16.45	17.02	n.d.
**pH**	–	7.30	7.44	7.02	7.11	6.96	7.14	6.94	7.07	7.15	6.98	7.31	8.2
**D.O.**	mg/L	2.15	2.76	1.71	3.09	1.87	2.14	2.25	2.39	3.26	2.32	3.23	n.d.
**Eh**	mV	181	188	146	143	183	3.0	193	150	143	190	−1	n.d.
**TOC**	mg/L	3.9	4.2	5.1	4.2	4.2	4.6	4.6	4.8	4.1	4.2	4.6	1360
**BOD**_**5**_	mg O_2_/L	3.2	6.5	7	4.5	3.8	4.5	6.9	5.0	4.4	6.5	6.3	450
**COD**	mg/L	5	7.5	7.5	5	5	7.5	7.5	7.5	5.0	7.5	7.5	2680
**Li**^**+**^	µg/L	7.7	6.3	4.6	4.5	4.2	4.6	6.9	7.8	2.7	3.9	8.1	31.1
**Na**^**+**^	mg/L	127.8	45.2	53.9	23.1	20.5	35.2	120.2	161.2	18.3	12.6	154.9	2538
**N-NH**_**4**_^**+**^	mg/L	<0.1	<0.1	<0.1	<0.1	<0.1	<0.1	<0.1	<0.1	<0.1	<0.1	<0.1	2009
**K**^**+**^	mg/L	3.2	1.6	2.1	4.7	3.7	2.2	2.1	4.2	1.1	3.2	4.2	1807.2
**Ca**^**2+**^	mg/L	106.5	120.2	101.3	91.9	110.3	106.6	127.1	121.6	115.0	96.1	110.3	31.5
**Mg**^**2+**^	mg/L	60.8	36.2	54.1	48.8	47.8	52.0	65.1	69.3	50.4	47.3	61.4	40.7
**Cl**^**−**^	mg/L	261.5	112	106.5	41.3	46.9	74.0	250	339.8	123.8	26.6	287.7	3286.5
**F**^**−**^	mg/L	<0.1	0.2	0.2	0.2	0.2	0.2	0.2	0.2	0.2	0.1	0.2	2.7
**SO**_**4**_^**2−**^	mg/L	32.3	12.5	15.2	13.7	15.2	20.0	34.2	45.6	5.6	11.4	41.8	10.6
**HCO**_**3**_^**−**^	mg/L	445.5	414.9	476.0.	457.7	488.7	488.7	506.5	512.6	396.6	476.0	506.5	12,081.96
**NO**_**3**_**-**	mg/L	32.4	51.2	27.5	20.6	38.5	36.3	36.7	30.0	39.7	21.4	17.1	2.70
**Fe**^**2+**^	µg/L	7.4	21.4	12.5	2.9	8.0	329.5	9.7	13.5	136.7	8.3	624.2	3696.0
**Mn**^**2+**^	µg/L	0.3	2.6	0.4	0.5	0.7	7.3	0.2	0.6	6.0	0.6	10.5	23.0
**Tritium**	TU	n.d.	n.d.	2.4±0.72	n.d.	2.50±0.71	2.10±0.55	1.70±0.55	2.00±0.89	3.00±0.72	n.d.	2.00±0.69	225±11
**d-excess**	(‰)	n.d.	n.d.	14.57	n.d.	15.09	15.57	14.63	14.56	14.04	n.d.	14.60	47.61

The data contains the land use analysis and the estimation of nitrogen contributions deriving from fertilizers.

The landfill group is located in the municipality of Conversano, close to Bari, the main town in the Apulia region, South Italy. Table 1 shows the sampling point used in this study. The study area is located in the largest coastal and karstic aquifer of Italy [2], [3], widespread hit by seawater intrusion effects [4].

## 2 Materials and methods

The pH, Electrical Conductivity (EC), Temperature (T), Redox Potential (Eh), and dissolved oxygen (DO) were measured in the field by means of a multiparametric probe (Table 2, Table 3, Table 4). The total alkalinity was determined by titration with 0.1 N HCl to a pH of 4.5. Samples for cation analysis were preserved by the addition of HNO_3_ to a pH < 2.

Water samples for metals determination was not filtered but was acidified before chemical analysis. This choice was due to the scope to explain some anomalously high iron contents that were detected in previous determinations in the area. The latter fraction was filtered to tap suspended particles from filters to be studied with the scanning electron microscopy. The scope of both fractions was to verify if they can explain a dissolved metal enrichment in the case of sampling without filtering before acidifying.

Water chemistry analyses were carried out at the chemical laboratory of DiSSPA. Anions (F^−^, Cl^−^, NO_3_^−^, SO_4_^2^) and ammonium ion (NH_4_^+^) were analyzed by ion chromatography, while Li^+^, K^*^, Na^+^, Ca^2+^, Mg^2+^, Fe and Mn by means of ICP-OES spectrometry (Table 2, Table 3, Table 4).

The charge balance errors for the analyses was mandatory within 5%.

The multi-isotope characterization of groundwater and leachate was focused on δ^18^O, δ^2^H, ^3^H, δ^13^C, δ^15^N-NO_3_^−^ and *δ*^18^O-NO_3_^−^ [1].

It could be suggested a refrigerated storing of the polyethylene bottles of tritium (^3^H) water samples until the laboratory analysis starts.

Groundwater and leachate ^3^H level was determined at the Hydroisotop Schweitenkirchen GmbH Laboratory (Germany), by liquid scintillation counting (LSC), proceeded by electrolytic enrichment. Due to the low level of tritium in groundwater, each groundwater sample was subjected to an electrolytic enrichment process before the measurement (Table 2, Table 3, Table 4).

**Fig. 4:**
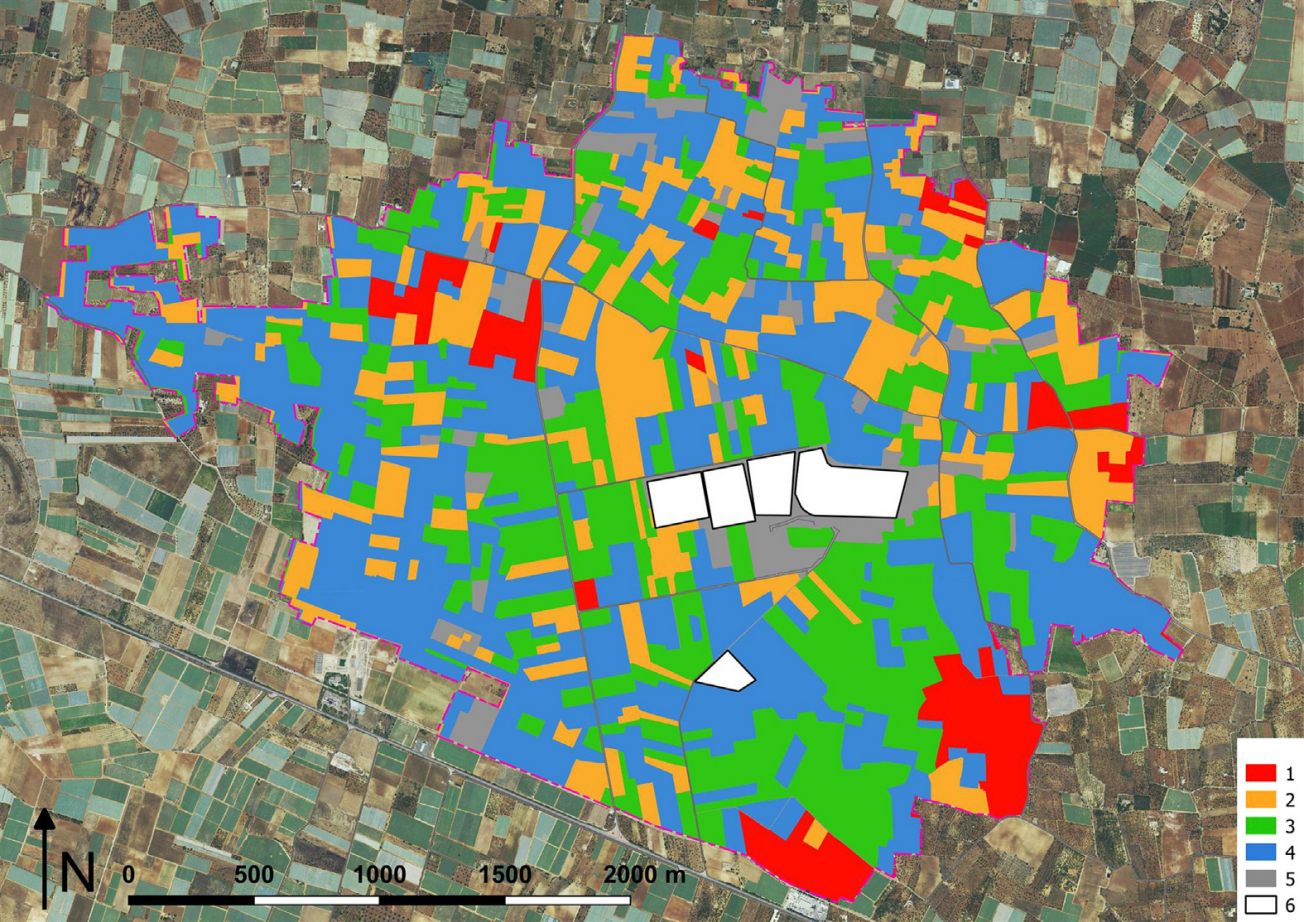
Map of main cultivation types close to landfills. Legend: 1) Orchard; 2) Arable; 3) Olive groves; 4) Vineyard; 5) Other; 6) Landfill. Cultivation areas are in Table 5.

The land use analysis pursues the characterization of nitrogenous sources due to fertilizer use, working on remote sensing maps, refined by on site GPS observations, and technical interviews (Fig. 4). On this basis, it is possible to recognize cultivation (Table 5), acquiring data on fertilizer loads.

**Table 5:** Main cultivation types and areas close to the group of landfills (Fig. 4). Total area 7.86 km^2^.

**Crops**		**Area (km**^**2**^**)**	**Area (%)**
1	Orchard	0.51	6.49
2	Arable	1.56	19.85
3	Olive groves	2.10	26.72
4	Vineyard	3.40	43.26
5	Other	0.29	3.38

Rainfall and temperature data were automatically stored using a gauge installed inside the study area (Fig. 1, Table 6).

**Table 6:** Monthly rainfall and air temperature measurements. (*) Due to seven days of data missing, monthly values of December 2014 were estimated using the closest regional gauge (distance 4.5 km).

Year	Jan.	Feb.	March	April	May	June	July	Aug.	Sept.	Oct.	Nov.	Dec.
Rain (mm)												
2014	30.6	27.2	23.8	103.2	54.0	84.2	100.2	8.6	64.2	66.2	50.0	38.6*
2015	108.0	71.6	115.0	25.8	41.2	45.0	0.0	1.4	44.8	101.2	78.2	9.2

Temperature (°C)												
2014	8.8	9.8	9.9	12.3	15.4	20.3	21.7	23.0	18.5	15.3	13.0	9.4*
2015	6.8	6.6	8.9	12.0	17.6	20.3	24.6	23.1	20.2	14.8	10.1	7.2

Using a water-level tape, piezometric depth measurements were realized in some wells, where it was possible to remove pumps and pumping tubes (Table 7).

**Table 7:** Piezometric survey (Nov. 15, 2016), measurements of piezometric depth below ground altitude (Table 1).

**Well**	**Depth to piezometric level (m)**
11	126.78
12	124.04
13	121.73
14	121.28
15	120.47
19	137.20
