# Supplementary material for: Data to clarify the landfill role in the case of groundwater quality degradation (Southern Italy)
Source: Data Brief. 2018 Sep 7;20:1489–99. doi: 10.1016/j.dib.2018.08.201 (PMC6153389; doi:10.1016/j.dib.2018.08.201)
Supplement: Supplementary file 1 — Transparency document [file mmc1.doc]

**Bari, Italy**

**May 24, 2018**

Dear Editor:

I would like to submit our manuscript entitled “Data to clarify the landfill role in the case of groundwater quality degradation (Southern Italy)” by Livia Emanuela Zuffianò, Pier Paolo Limoni, Giorgio De Giorgio, Maurizio Polemio for publication in “*Data in Brief*”.

Declarations of interest: none

Dr. Maurizio Polemio

m.polemio@ba.irpi.cnr.it
